# Study on stage division and evaluation index of microwave deicing technology for pavement concrete

**DOI:** 10.1038/s41598-025-13126-9

**Published:** 2025-07-30

**Authors:** Tao Chen, Ying Zhang, Biao Ren, Yan Lv, Tao Ge, Erlei Bai, Li Wang

**Affiliations:** 1 Faculty of Engineering, Huanghe S&T University, Zhengzhou, 450063 Henan China; 2 Aeronautics Engineering College, Air Force Engineering University, Xi’an, 710038 Shaanxi China

**Keywords:** Pavement concrete, Microwave deicing technology, Carbon fiber, Evaluation index, Stage division, Civil engineering, Structural materials

## Abstract

Microwave deicing technology has received growing attention as one of the deicing solutions for road surface icing in cold climates due to its benefits, such as energy savings, environmental protection, high efficiency, pavement protection, and others. In this study, practical and theoretical research was conducted on the stage division of the physical process of microwave deicing of road surface concrete and the deicing technology assessment index. Based on the experimental results and the characteristics of ice melting, the microwave deicing process can be divided into four stages, respectively: Heat from Concrete Stage (HCS), Heat from Water Stage (HWS), Ice Thinning Stage (ITS), and Ice Breaking and Melting Stage (IBMS). Five indicators may be used to evaluate the deicing efficiency of road surface deicing technology: the time when the temperature of the concrete surface reaches 0 ℃, the time when the water layer is fully formed, the time spent from the complete formation of the water layer to ice breaking, the deicing area and the ice breaking area. The microwave deicing efficiency of road surface concrete can be improved by combining carbon fibers in the concrete, and the enhancing impact varies with carbon fiber length. When the length of the carbon fiber is 0.6 cm, the deicing speed of the concrete specimen is 2.0 times faster than that of the concrete specimen without the carbon fiber, and the deicing area is 1.2 times larger.

## Introduction

In the winter, freezing on the road surface causes traffic congestion and travel disruption, demanding rapid deicing to restore normal and safe traffic conditions^1^. As a result, in cold regions, road surface rapid deicing technology is a common and pressing issue, especially in highland alpine environments^2,3^. There are many traditional deicing technologies, mainly physical deicing, chemical deicing, and thermal deicing^4^.

Physical deicing is divided into manual deicing and mechanical deicing. The former uses artificial tools such as spades and shovels to remove snow cover on the road. This method has the advantages of simple technology and a high removal rate, but it is inefficient and consumes a lot of manpower. It is only suitable for removing ice and snow on a small range of pavement. The latter uses mechanical devices to remove the ice and snow on the pavement. However, the pure mechanical deicing method has a low net removal rate, and the crushing force of the mechanical device is not easy to control. If the crushing force is too high, it will damage the pavement and destroy the pavement signs. The removal rate is low if the crushing force is too low^5^.

Chemical deicing is achieved by adding chemicals to the water to lower the water’s vapor pressure and the melting point of ice and snow, thereby dissolving the ice and snow and ultimately clearing the road of ice and snow^6^. Chemical deicing technology is a common means of road deicing, but chemical agents have a specific corrosive effect on the environment, roads, and vehicles.

Thermal deicing is carried out by transferring chemical energy, electric energy, solar energy, geothermal energy, and other energy to the concrete pavement through a particular medium or by directly heating it and different energy to the concrete pavement through a specific medium or by directly heating it. This method has a high net removal rate. Still, the simple thermal deicing technology often consumes serious energy, and the energy utilization rate is not high because the heating is blind, the heat conduction takes a specific time, and the medium will dissipate part of the energy. In addition, some thermal deicing needs to lay heat pipes in advance, and the maintenance process is complex.

Pavement microwave deicing technology is a technology that uses microwaves to heat the pavement to melt the ice layer and combine with mechanical devices for deicing. Microwave is a kind of electromagnetic wave with high frequency. The reflection and absorption of microwaves are selective. When encountering metal objects, a large number of reflections will be generated. When encountering insulating objects, most will be transmitted and continue to propagate in the insulator, resulting in loss and heating^7^. The propagation law of microwave in the medium is affected by the dielectric constant of the medium, which can be described by the ratio of the imaginary part and the real part of the complex dielectric constant. This ratio is named the loss tangent because it reflects the energy that is converted into heat energy and consumed in unit volume and unit time.

$$\tan \delta =\varepsilon ^{\prime\prime}/\varepsilon ^{\prime}$$

Microwave radiation applies alternating electromagnetic fields with a high frequency, in the order of megahertz, causing a change in the orientation of polar molecules, which results in internal friction and increases the material temperature^8^. The loss tangent of ice is very small and can be almost ignored. Therefore, when microwave irradiates the concrete pavement with ice accumulation, it can directly irradiate the concrete through the ice layer, make the concrete heat and melt the ice layer, weaken the bonding force between the ice layer and concrete, and achieve the effect of deicing.

Microwave deicing technology has successfully overcome the limitations of traditional deicing methods and has broad application prospects^9,10^. Compared with simple mechanical deicing technology, this method greatly reduces the mechanical damage caused by mechanical deicing technology to the pavement. Compared with chemical deicing technology, microwave deicing technology is not corrosive to the pavement and is more environmentally friendly. Compared with the deicing technology of external heating, microwave heating starts from the bottom of the ice layer, so its net removal rate is very high. In addition, microwave heating is volume heating, with small thermal inertia and uniform heating, which overcomes the problem of temperature stress on the pavement caused by an excessive heating gradient of the thermal blowing method. In addition, microwave heating is selective heating, which overcomes the problem of low energy utilization of the thermal blowing method. Finally, microwave heating directly separates the ice layer from the pavement and eliminates the adhesion of the ice layer through the change of phase. Therefore, it also has high deicing efficiency for the ice layer formed by freezing rain and ice accumulation. In short, as a highly efficient, practical, economical, low consumption, green, and compatible deicing method, microwave deicing technology has attracted the attention, research, and development of scholars in recent years.

Researchers have identified and unified the mechanism of this physical process^11,12^. However, in both laboratory and theoretical studies, there are still certain research challenges. After a lot of research and analysis, it can be found that the difficulties can be summarized into five aspects.

Firstly, the ice preparation process is quite complex. When preparing the pavement concrete specimen covered with ice in the laboratory, in order to condense the liquid water into ice, the means of freezer cooling can generally be used. However, because the concrete surface is frequently uneven, many tests must quantitatively control ice thickness in order to account for the impact of ice thickness on deicing performance. As a result, preparing the mold for the water layer is tough. Microwave heating of concrete specimens is currently more widespread^13^, but microwave deicing of concrete ice layer research is less common. The temperature rise law of the ice layer can be somewhat reflected by the microwave heating law without the ice layer. However, the temperature rise law of concrete specimens with ice layers is actually much more complex due to the phase transition of ice and water, as well as the influence of water on microwave absorption and heat transfer.

Secondly, traditional temperature sensors are made of metal, which makes them ineffective for measuring the temperature of microwave heating because microwaves interfere with the signal of metal temperature sensors, whereas an infrared temperature measurement system can only measure the temperature of the ice layer^14^. The temperature of the concrete surface and the ice contact surface is difficult to obtain in laboratory studies.

Thirdly, microwave deicing is a multi-phase, multi-disciplinary procedure^15^. The complete theories of heat conduction, composite materials, and electro-magnetism are all used to interpret the mechanism. As a result, the theoretical solution for the rate of temperature rise is more complicated^16^.

Fourthly, microwave deicing is a physical process that involves the interaction of microwave and thermal fields. Its mathematical description is a transcendental equation, which can only provide an approximate solution through simulation rather than an analytical answer^17,18^.

Fifthly, for the use of open microwave sources, the safety of microwave leakage should be fully considered. Microwave leakage may not only waste energy but also threaten human health and damage all organic systems nearby. Scientists have a common concern about the safety of open microwave sources. When the deicer works, it must meet the relevant national sanitary standards for microwave radiation. In the experimental research, it is necessary to test the specific degree of microwave leakage.

From the above analysis, it can be seen that in the process of microwave deicing technology, from experimental research to practical application, there are two important scientific issues that need to be solved. One is the stage division of the physical process of microwave heating and melting ice, and the other is the determination of the evaluation index of deicing technology. Therefore, in order to accelerate the promotion of microwave deicing technology, this paper prepares different kinds of concrete specimens and carries out experimental research on microwave deicing of concrete with ice layers. According to the experimental results, the temperature rise rate of the microwave deicing process is divided into heat dissipation rate and heat production rate, and the theoretical formula is deduced. Finally, four indexes of the physical process of microwave deicing and five evaluation indexes of microwave deicing technology are put forward. The conclusions obtained can have a certain theoretical guiding significance for experimental research in the field of pavement deicing and promote the practical application of pavement microwave deicing.

## Research methods

### Test specimen

**Fig. 1:**
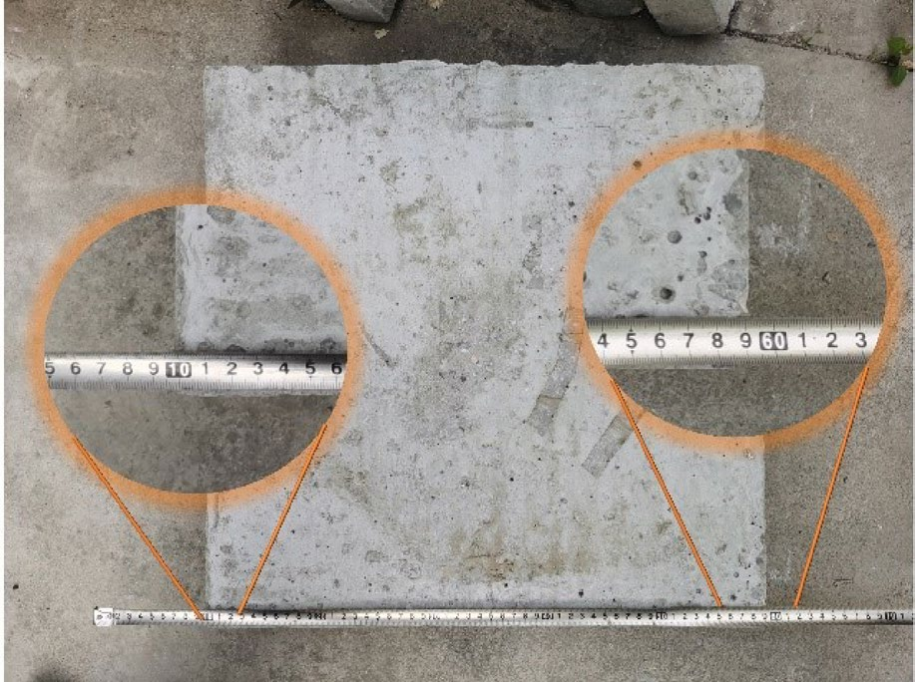
The size of cured concrete specimens.

In order to expand the temperature rise range of the experiment and better describe the law of wave absorption and deicing of pavement concrete, three groups of carbon fiber reinforced control experiments are designed on the basis of ordinary concrete. Carbon fibers (Toray T700SC, diameter 7 μm, density 1.8 g/cm³) were incorporated at 0.6% volume fraction. Fiber lengths (0.1 cm, 0.3 cm, 0.6 cm) were selected based on optimal dispersion and microwave absorption. To comprehensively study the microwave deicing performance of pavement concrete, carbon fibers with lengths of 0.1 cm, 0.3 cm, and 0.6 cm were added into the concrete to obtain three groups of carbon fiber modified concrete, numbered 0.1CFC, 0.3CFC, and 0.6CFC, respectively. Based on improving its absorbing efficiency, the law of absorbing and deicing is further studied. That is, four groups of specimens with a carbon fiber content of 0%vol, 0.1%vol, 0.3%vol, and 0.6%vol were poured, respectively. Specimen nomenclature: PC (Plain Concrete), 0.1CFC (0.6% vol 0.1 cm fibers), 0.3CFC (0.6% vol 0.3 cm fibers), 0.6CFC (0.6% vol 0.6 cm fibers). The 0.6% vol rate was empirically determined to maximize microwave absorption while maintaining concrete integrity—lower rates showed negligible effects, while higher rates caused workability issues^7^. The size of the test piece is 50 cm×50 cm×5 cm, as shown in Fig. 1.

### Ice preparation

At present, there is much research on the micro-wave deicing technology of pavement concrete, but due to the difficulty of ice preparation, such as the ice thickness is not easy to grasp, the ice-making mold should be strictly sealed before ice-making to ensure that the water layer is not lost, and it should be able to better remove the mold after ice making, or conduct experiments directly. In this experiment, an expandable polyethylene (EPE) shockproof pearl cotton foam board was used to make the mold. It not only ensures water retention and ice thickness but also makes the mold with this material easy to form, low density, and easy to move. In addition, compared with rigid materials, the foam board has a strong deformation ability, which can avoid the damage caused by the ice volume expansion and the uneven ice surface in the ice-making process. Most importantly, this material will not reflect microwaves, so it will not affect the test results of microwave irradiation.

The inner height of the mold is 65 mm, 15 mm higher than the specimen. Inject the water layer and control its thickness. A centimeter scale was made from cardboard and glued to the edge of the foam mold to control the thickness of the water layer, after which a large amount of test water was injected. In this paper, the thickness of the water layer was controlled at 10 mm, so the water injection was stopped when the water layer reached the 10 mm scale. After the water layer solidified, the thickness of the ice layer was recorded.

### Experimental equipment

**Fig. 2:**
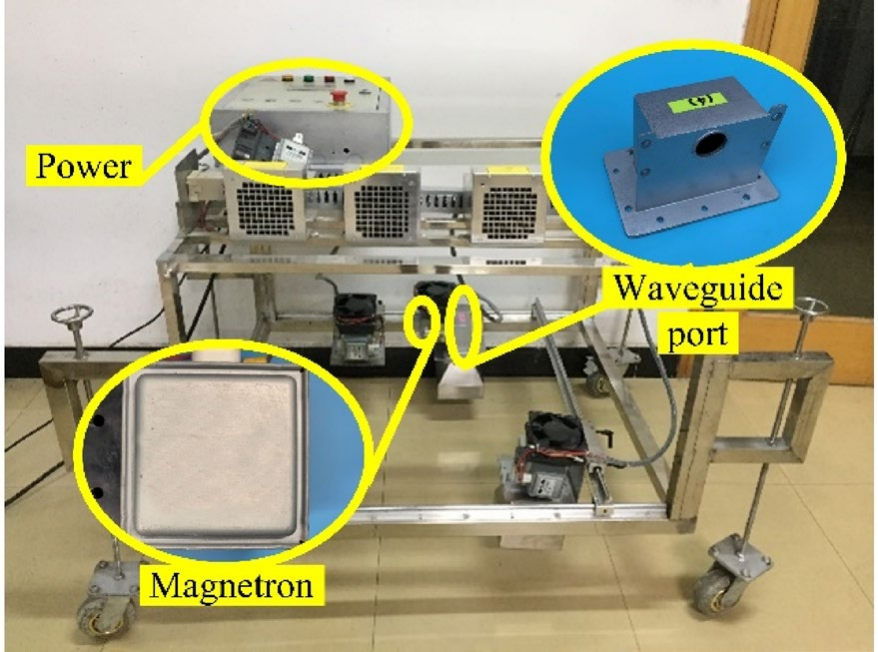
Open wave source microwave deicing equipment.

In order to simulate the actual deicing conditions as much as possible, the open microwave deicing technology is adopted in the test. Different from closed microwave deicing devices such as microwave ovens, open wave source microwave deicing equipment (Fig. 2) is independently designed and produced by Shanghai Saihan mechanical equipment.

The microwave is excited by a magnetron. Magnetron is an electric vacuum device that uses electrons to complete energy conversion in a vacuum, which can obtain high power. After the microwave is excited from the magnetron, it is transmitted and irradiated through the waveguide. The waveguide is a kind of microwave transmission line. For microwave transmission systems with a small transmission distance, the microwave transmission line is often used. The material of the waveguide is a 2 mm thin wall made of alloy aluminum. In order to ensure more propagation of microwave energy, the size of the waveguide port needs to be determined according to the frequency of the wave. After the microwave passes through the waveguide port, it still needs to be conducted through the horn port to irradiate the ice surface and act on the concrete to raise the concrete temperature to achieve the effect of deicing.

**Fig. 3:**
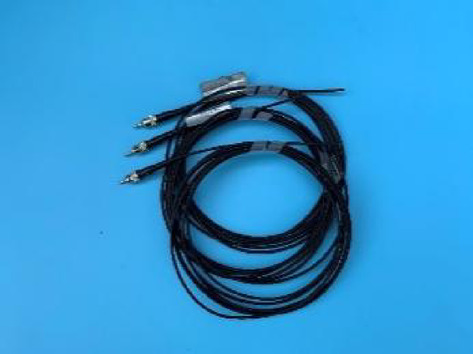
Optical fiber sensor.

When open microwave irradiation is adopted in the field test, the amount of microwave radiation must meet the relevant requirements of “Hygienic standard for microwave radiation in the Workplace (GB 10436-89)”. Specimens were centered 20 cm below the waveguide outlet (Fig. 3), ensuring uniform microwave exposure per GB 10436-89. For this, we conducted a special test in the experiment. The results show that under the irradiation of a 2.45 GHz microwave source with a power of 1 kW, no microwave signal can be measured beyond 3 m. This shows that the safety of “open wave source microwave deicing equipment” is reliable.

**Fig. 4:**
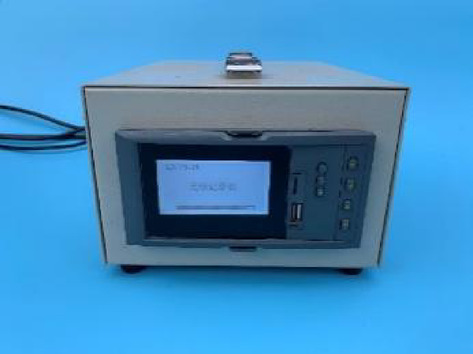
Paperless recorder.

Furthermore, to avoid microwave interference with the temperature sensor, the standard metal temperature sensor is not employed in the test; instead, an optical fiber sensor for temperature measurement is chosen. The optical fiber sensor is a YL-PL passive optical fiber temperature sensor (Fig. 3), which is used to measure the temperature of a fixed point on the upper surface of the concrete specimen. The paperless recorder is used to store the temperature data (Fig. 4).

### Experimental process

**Fig. 5:**
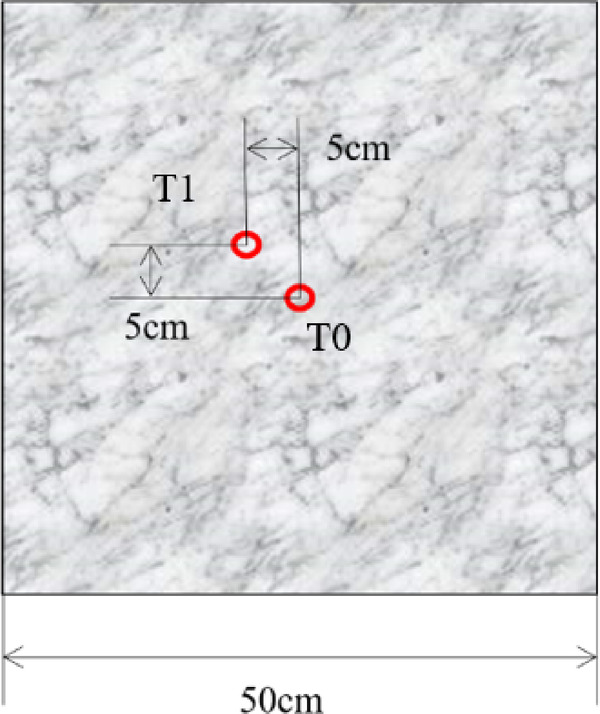
Schematic diagram of temperature measurement center point T0 and edge point T1.

- Fix the optical fiber sensor at the center point (T0) and edge point (T1) of the test piece, and test the temperature rise curve of both of the two points. The relative positions of T0 and T1 are shown in Fig. 5.
- The open wave source microwave deicing equipment is used for microwave irradiation, and the optical fiber sensor records the real-time data of the changes of two temperature measuring points, T0, and T1 per second, so as to obtain the temperature rise curves of the center point and edge point of four groups of specimens.
- After microwave heating for 120 s, take the deicing effect picture. Then, the broken ice is knocked on with a small hammer and processed with CAD drawing software to obtain its deicing area and shape.
- Each group of test pieces shall undergo 5 cycles of ice layer preparation and microwave deicing, and the mean value shall be taken after eliminating the obvious bad value.

**Fig. 6:**
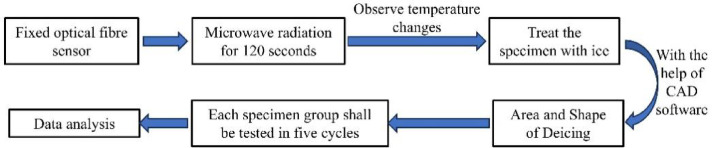
Flowchart of the study.

The flow chart of the study is shown in Fig. 6.

## Results

### Control group

**Fig. 7:**
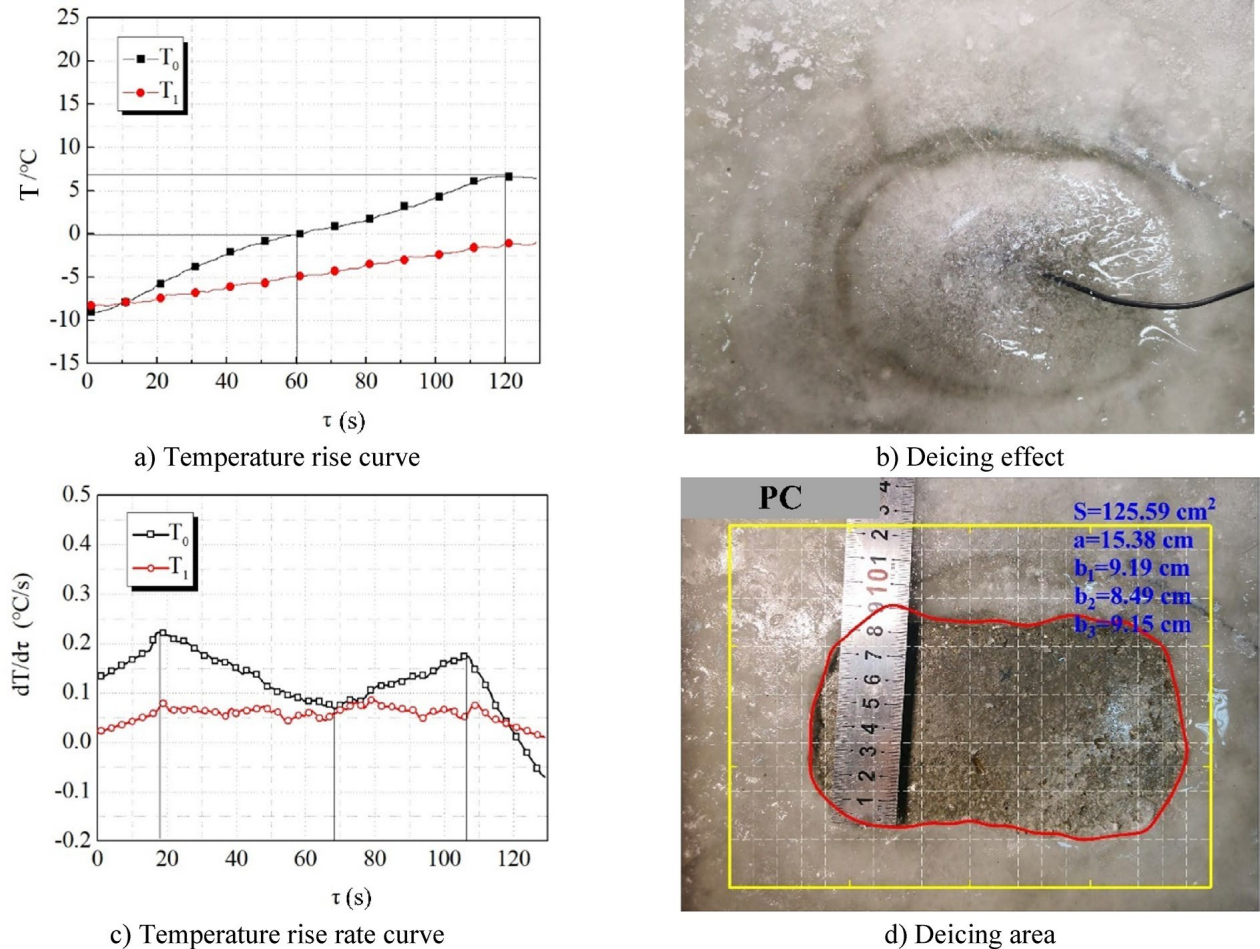
Wave absorbing deicing efficiency of PC.

**Fig. 8:**
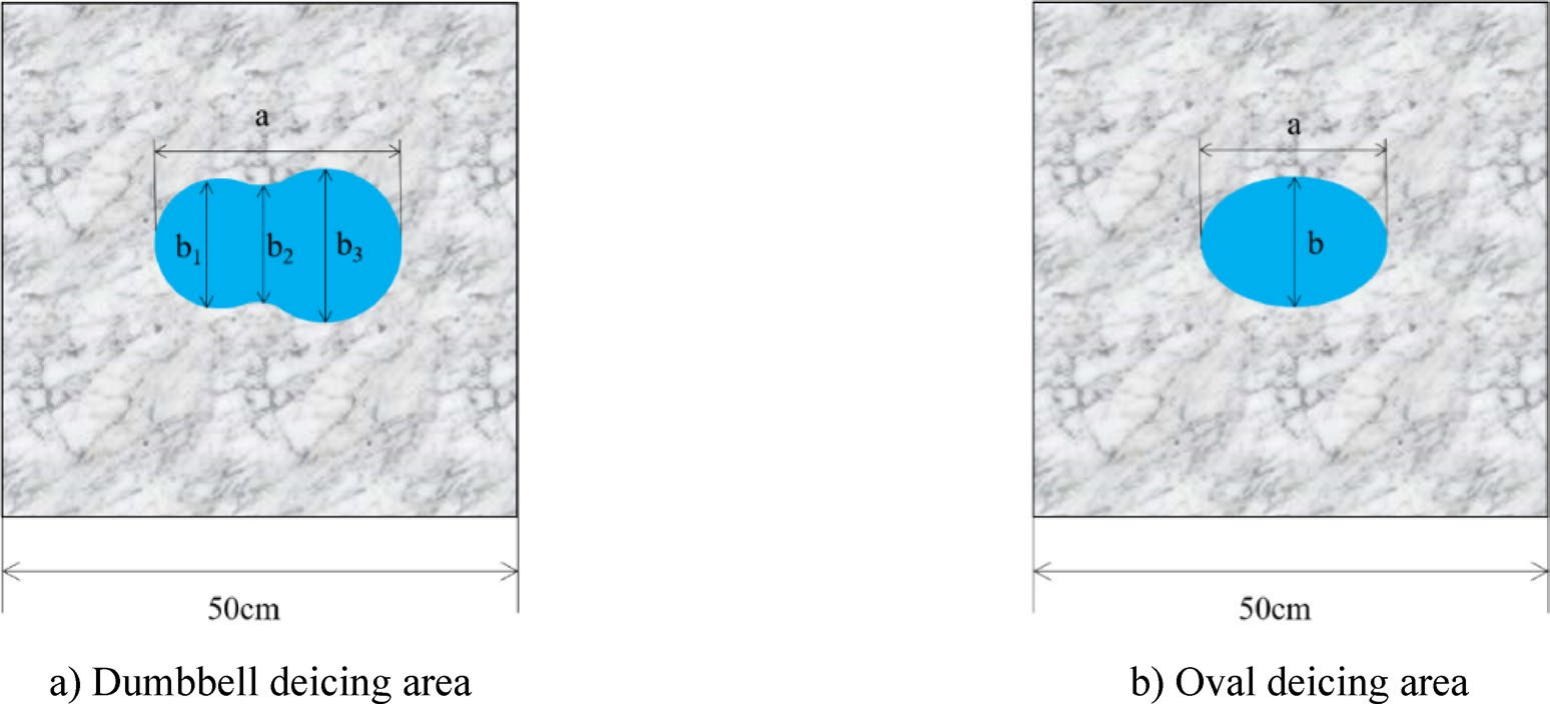
Schematic diagram of deicing area shape.

Firstly, we conducted the microwave irradiation test of overlying ice on PC as the control group. Figure 7a is the temperature rise curve of the center point T0 and edge point T1 of the concrete surface. Figure 7b shows the effect of deicing after microwave irradiation. Figure 7c is the temperature rise rate curve derived from the temperature rise curve. When the temperature rise rate curve is lower than 0, it indicates that the temperature decreases. Figure 7d is the calculation diagram of the deicing area. The closed curve surrounded by the red line is the outline line of the deicing area. Letter S in the figure represents the deicing area. When the deicing area is dumbbell-shaped dumbbell shaped, the four parameters of the dumbbell-shaped deicing area, centerline length a, left semicircle width b1, centerline width b2, and right semicircle width b3, are used to describe the shape of the deicing area (Fig. 8a). When the deicing area is elliptical, two parameters, centerline length a and centerline width b, are used to describe the deicing area shape (Fig. 8b).

From Fig. 7, it can be seen that:

(1) When heated for 10 ~ 20 s, the temperature rise rate increases gradually. During this time, the microwave bypasses the ice and directly heats the concrete. When there is no ice on the surface of the specimen, the concrete needs to conduct heat convection to the air at a lower temperature, while when there is ice on the surface of the specimen, the concrete surface needs to conduct heat conduction to the ice. Therefore, the concrete temperature increases gradually, while the ice temperature remains unchanged, and the temperature difference decreases gradually, which leads to the temperature gradient decreasing and the heat dissipation rate decreasing.

(2) After heating for 60 s, the temperature at the central point T0 reaches 0 ℃, and the temperature rise rate decreases gradually with the increase of the concrete surface temperature, and reaches the minimum when the temperature reaches 0 ℃. This is because there is no liquid water between the ice layer and the concrete before 0 ℃. According to the boundary conditions of heat conduction, the temperature of the lower surface of the ice layer is equal to the temperature of the upper surface of the concrete, so the temperature difference on both sides of the ice layer becomes larger and larger. The faster the heat dissipation rate is, the slower the temperature rise rate is when the heat production rate is certain.

(3) After the temperature at the center point T0 reaches 0 ℃, the temperature rise rate increases gradually, which is because the melting of the ice layer on the concrete surface gradually produces liquid water. Water has a greater ability to absorb microwaves and a greater heat generation rate. On the other hand, the ice water interface temperature is 0 ℃, the upper surface temperature of the ice layer is room temperature, the temperature difference on both sides of the ice layer is almost unchanged, and the thickness of the ice layer is also almost unchanged, so the temperature gradient is almost unchanged. According to Fourier’s law, the heat transfer rate is directly proportional to the temperature gradient, so the heat dissipation rate of the ice layer is almost unchanged, and the overall temperature rise rate increases to a certain extent.

(4) After heating for 108 s, the temperature rise rate decreases again. That is because the water layer is almost completely formed, while the heat production rate is nearly unchanged or rises slowly, and the thickness of the ice layer decreases sharply. When the ice is about to break, the thickness of the ice layer approaches 0, and the temperature gradient approaches infinity. Therefore, the heat dissipation rate is great for a period of time before the ice breaks, and the ice layer absorbs a lot of heat until the ice melts. The time of this stage is very short, only about 10 s, and the ice-breaking area on the ice surface in the deicing effect diagram (Fig. 7b), and the area is very small. (5) The final deicing area of PC is small, which is 125.59cm^2^, and the deicing shape is close to the ellipse (Fig. 7d). (6) The temperature rise curve reaches the maximum at 120 s, indicating that the temperature stops rising immediately after the heating is stopped. The microwave deicing technology has good real-time performance, small thermal inertia, and easy operation.

### Experimental group

**Fig. 9:**
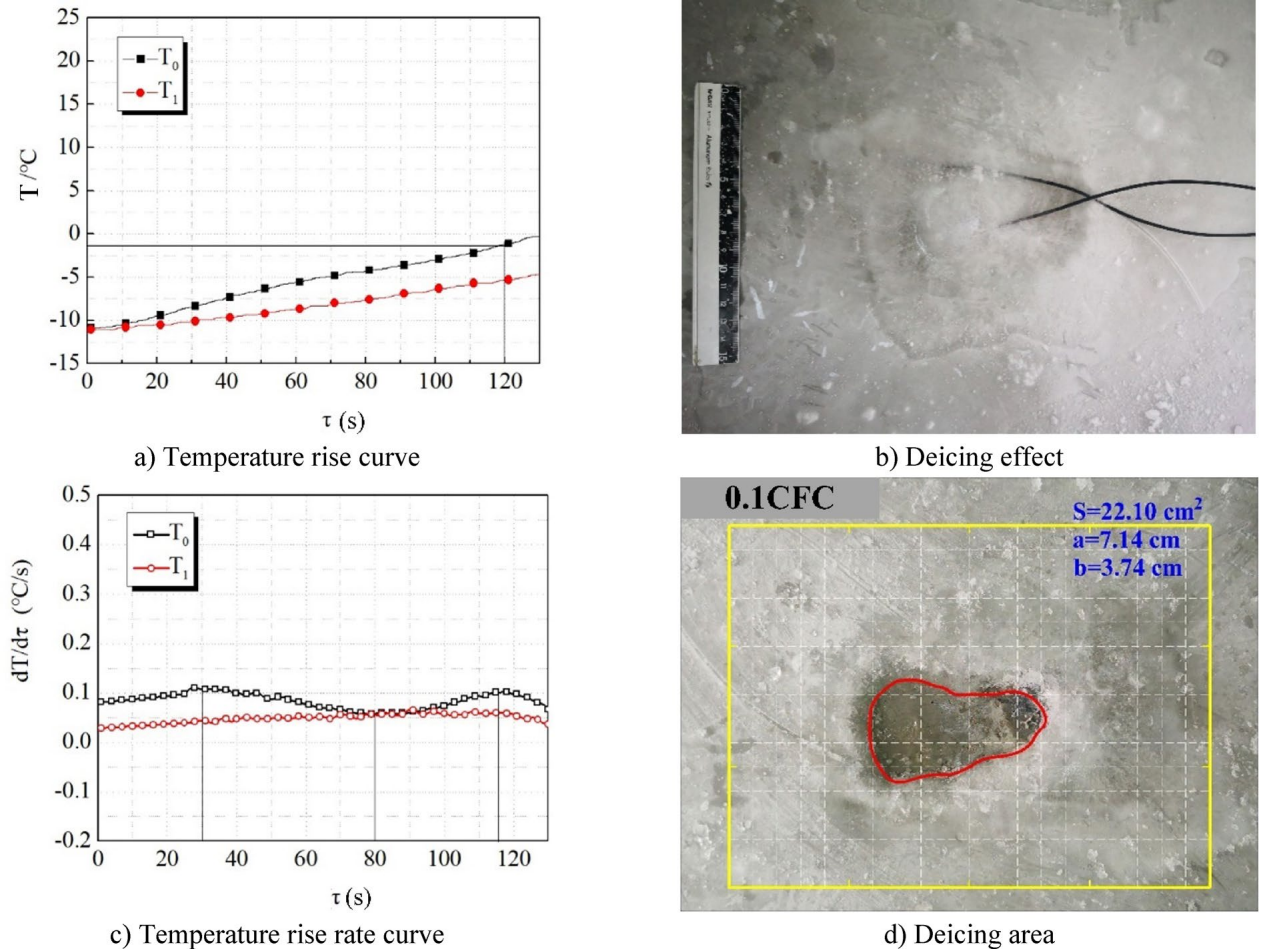
Wave absorbing deicing efficiency of 0.1CFC.

Figure 9 shows the efficiency of wave absorption and deicing of concrete specimens when the length of carbon fiber is 0.1 cm. From Fig. 9, it can be seen that: (1) The temperature rise rate of the temperature rise curve is very slow, the temperature at the central point T0 is always lower than 0 ℃ (Fig. 9a), the temperature rise amplitude is only 9.7 ℃, and the temperature rise rate is very low, which is between 0 and 0.1 ℃/s (Fig. 9b). It shows that the microwave absorption performance of 0.1CFC is poor. It improves the electrical conductivity of concrete because of the addition of carbon fiber. The reason is that the length of 0.1 cm is too short to disperse easily after mixing into concrete, resulting in the overlapping of carbon fibers into a ring or even a mass, forming a large area of conductive area, which is similar to adding small metal sheets into concrete. Therefore, there is a division between bad conductors and good conductors in the concrete. After microwave irradiates into the concrete, it is either absorbed by the original concrete matrix or reflected by a “small metal sheet,” which reduces its microwave absorption performance. (2) There is no ice breaking on the upper surface of the ice layer, and the deicing area is only 22.10 cm². The deicing range is very small. (3) A few ice layers in the center can be removed by knocking with a hammer (Fig. 9d), indicating that when the concrete surface temperature is lower than 0℃, the ice layer has not been separated from the pavement, and it is still difficult to remove ice.

**Fig. 10:**
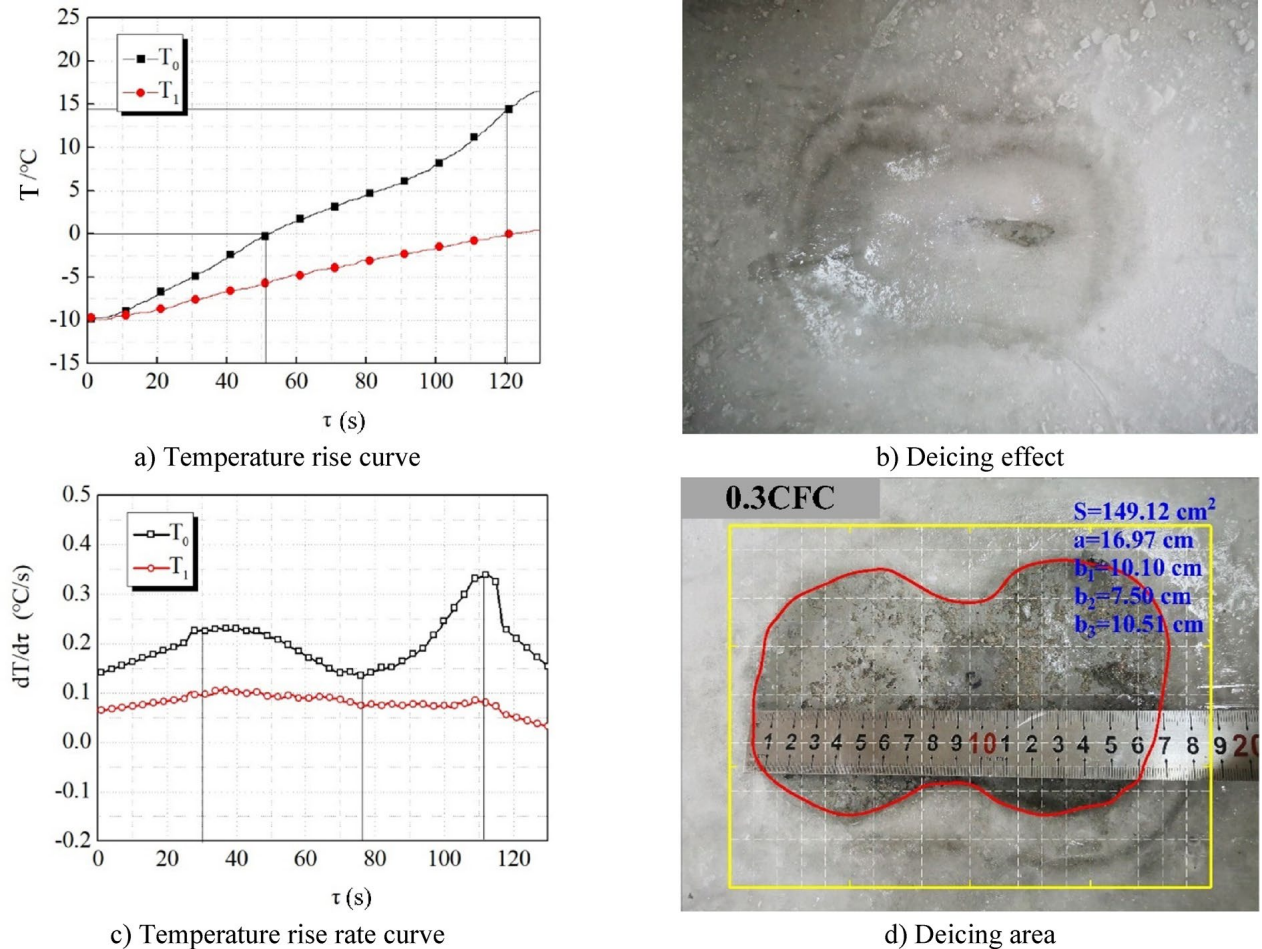
Wave absorbing deicing efficiency of 0.3CFC.

Figure 10 shows the efficiency of wave absorption and deicing of concrete specimens when the length of carbon fiber is 0.3 cm. From Fig. 10, it can be seen that: (1) The change law of the temperature rise curve of 0.3CFC specimen is consistent with that of PC. The temperature rise rate increases at the beginning and then decreases. After 0 ℃, the temperature rise rate increases significantly due to the emergence of liquid water (Fig. 10a & c). It should be noted that the temperature rise rate of the 0.3CFC specimen is slightly higher than that of PC in general. This is because the carbon fiber with a length of 0.3 cm has better dispersibility than the carbon fiber with a length of 0.1 cm. It is easier to disperse in the concrete rather than agglomerate, and it is more compatible with the concrete. This relatively uniform dispersion greatly improves the conductivity of concrete. In general, it directly improves the concrete from a bad conductor to a semiconductor, changes the dielectric constant of the composite, and improves its conductivity, so it improves its ability to absorb microwaves. (2) The temperature rise rate of 0.3CFC specimen after 0 ℃ is higher than that in the early stage, indicating that the area of the water layer is large and the wave absorption and heating rate is fast, which corresponds to its large deicing area in Fig. 9d. The deicing area is 149.12cm^2^, slightly larger than that of PC. (3) The temperature rise curve has an inflection point at 112 s (Fig. 10a), indicating that the ice layer on the surface of 0.3CFC specimen is the same as that on the surface of PC specimen, and ice breaking happens, which is consistent with the deicing effect diagram of 0.3CFC specimen (Fig. 10b). In general, the temperature rise law of 0.3CFC is similar to that of PC, and the deicing efficiency is slightly better than that of PC.

**Fig. 11:**
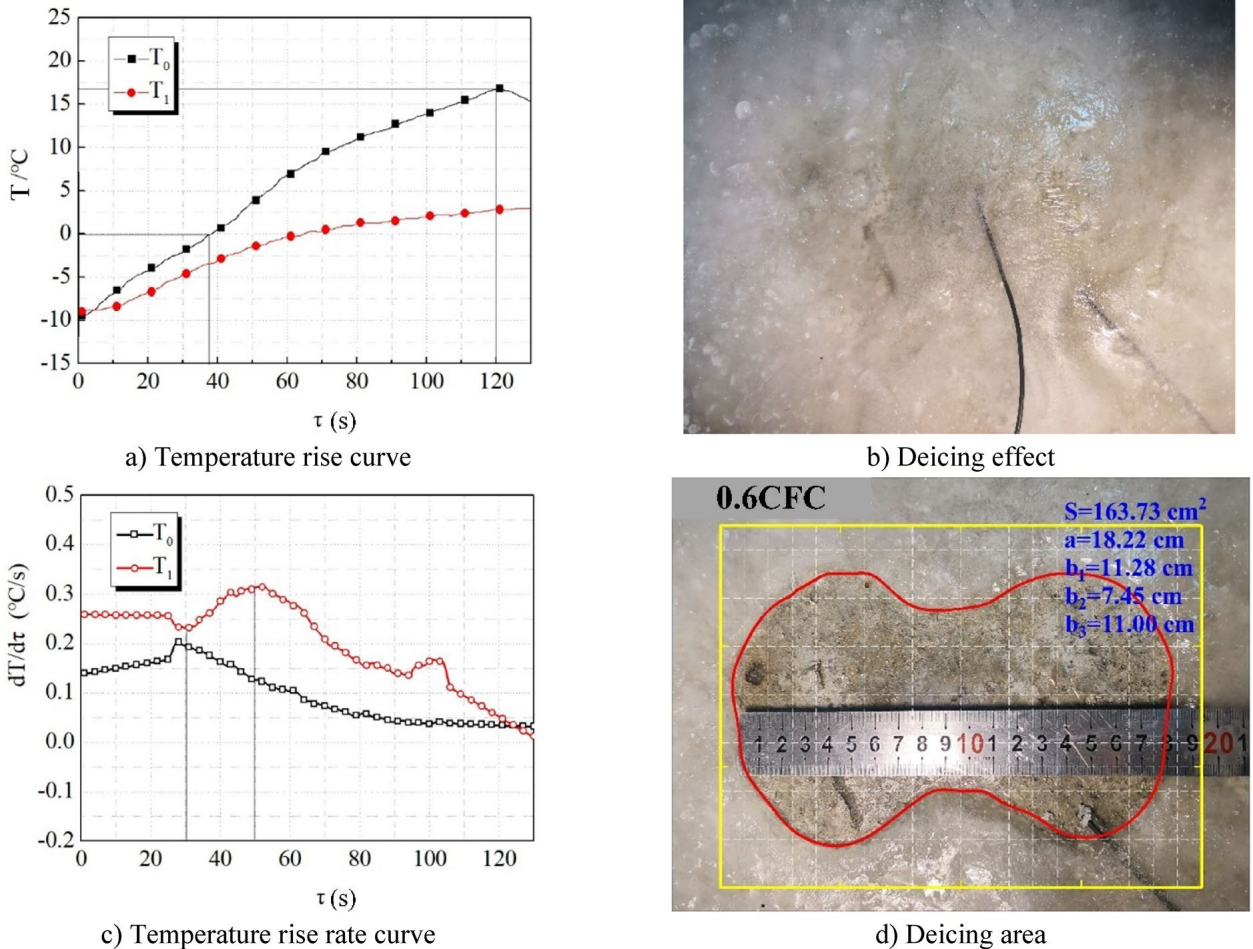
Wave absorbing deicing efficiency of 0.6CFC.

Figure 11 shows the efficiency of wave absorption and deicing of concrete specimens when the length of carbon fiber is 0.6 cm. From Fig. 11, it can be seen that: (1) before the temperature of T0 at the surface center point of 0.6CFC specimen reaches 0 ℃, the temperature rise rate curve remains horizontal (Fig. 11c), indicating that at this stage, the increase rate of heat production rate is similar to that of heat dissipation rate, so the temperature rise rate remains unchanged. (2) After the central point T0 of the specimen surface reaches 0 ℃, the temperature rise rate begins to rise, and the temperature rise rate decreases in a very short time (51s), indicating that the water layer has been formed. The thickness of the ice layer begins to decrease. (3) The temperature rise rate fluctuates around 100s. It can be seen from Fig. 11b that a layer of water film appears on the surface of the ice layer, indicating that the ice-breaking phenomenon is likely to bring fluctuations in the temperature rise rate. This is because the heat dissipation rate decreases abruptly at the moment of ice breaking so that the temperature rise rate will increase. With the further increase of the ice-breaking area, the heat dissipation rate will continue to rise, and the temperature rise rate will decline. (4) Overall, the temperature rise rate of the 0.6CFC specimen is much higher than that of PC. The carbon fiber with a length of 0.6 cm can not only be well dispersed into the concrete but also ensure a certain stiffness; it rarely overlaps or forms a ring with each other to enhance its microwave absorption performance.

## Theoretical analysis

### Four stages of microwave deicing

**Fig. 12:**
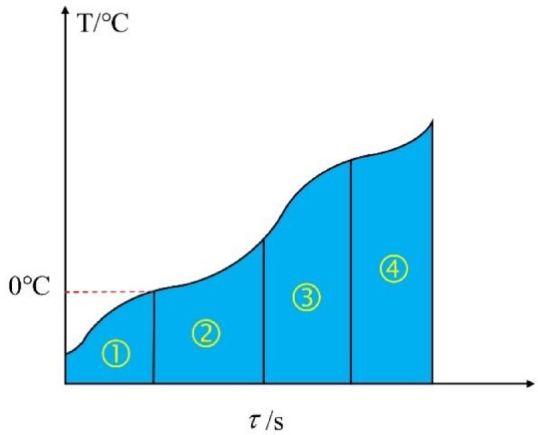
Schematic diagram of four stages of temperature rise curve.

Comparing the deicing effects of different groups of concrete specimens, the temperature rise curve can be roughly divided into four stages, which are shown in Fig. 12.

(1) The first stage is the Heat from the Concrete Stage (HCS). Concrete absorbs microwaves and generates heat. Then, the heat is dissipated to the ice layer and the interior of the concrete in the form of heat conduction. The heat generation rate and heat dissipation rate increase at the same time. Due to the low heat generation rate, the temperature rise rate of PC and 0.3CFC decreases before 0 ℃, while the temperature rise rate of 0.6cfc with a fiber length of 0.6 cm does not decrease significantly before 0 ℃.

(2) The second stage is Heat from Water Stage (HWS). Liquid water is gradually produced to replace the concrete to absorb the microwave as the heat source after the surface temperature of the specimen reaches 0 ℃, and the temperature rise rate increases. At this stage, the thickness of the ice layer is basically unchanged, and the absorbed microwave energy is transmitted laterally on the concrete surface, which gradually increases the area of the water layer. The formation time of the water layer of different specimens is different. To PC and 0.3CFC, it’s very long, which is because of their poor microwave absorption performance, while the specimens with the length of carbon fiber of 0.6 cm only maintained for about 10 s in the second stage, indicating that a large area of water layer has been formed at this time.

(3) The third stage is the Ice Thinning Stage (ITS). After the water layer is completely formed, the deicing area remains unchanged, and the thickness of the ice layer decreases, which is because the absorbed microwave energy is transmitted longitudinally to the ice layer. At the end of this stage, ice-breaking occurs gradually. At this stage, the temperature rise rate decreases rapidly due to the decrease in ice thickness.

(4) The fourth stage is the Ice Breaking and Melting Stage (IBMS). After ice breaking, in a small range of ice-breaking parts, the water layer is in direct contact with the air. The heat dissipation form changes from the heat conduction between the water layer and the ice layer to the thermal convection between the water layer and the air, which leads to a significant reduction in the heat dissipation rate. Therefore, the temperature rise rate will rise in a short period of time after ice breaking. On the other hand, the ice in other parts will also accelerate the rate of heat absorption, which leads to the alternating increase and decrease of temperature rise rate. It can be predicted that after the ice is completely broken and melted, the temperature rise rate will remain at a constant value, which is similar to the temperature rise law of microwave heating without overlying ice.

The four stages appear in chronological order. Considering that “the ice layer is completely separated from the pavement and the water layer is completely formed” exceeds the requirements of actual construction^19^, the best time for deicing should be in the second stage, which is after the water layer appears and before the ice breaking appears. In terms of temperature, that is after the temperature reaches 0 ℃ and before the temperature rise rate decreases.

### Formula derivation into four stages

According to the division of four stages, theoretical analysis is carried out for each stage.

The microwave deicing process of carbon fiber reinforced concrete can be divided into four stages chronologically: Heat from Concrete Stage (HCS), Heat from Water Stage (HWS), Ice Thinning Stage (ITS), and Ice Breaking and Melting Stage (IBMS). For any point in the space, the temperature rise rate is equal to the heat generation rate minus the heat dissipation rate^20^, that is:

$$\frac{{\partial {T_s}}}{{\partial \tau }}=\frac{{0.566 \times {{10}^{ - 10}}}}{{c\rho }}f{\varepsilon ^{\prime\prime}_{eff}}E_{{rms}}^{2}+\frac{\lambda }{{c\rho }}{\nabla ^2}{T_s}$$

In this formula,

$\frac{{\partial {T_s}}}{{\partial \tau }}$ is the derivative of temperature with time, that is, the rate of temperature rise.

${\nabla ^2}{T_s}$ is the derivative of temperature with space, that is, the temperature gradient.

*c* is the specific heat capacity, its unit is${\text{kJ/(kg}} \cdot {\text{K)}}$.

$\rho$ is density, its unit is${\text{kg/}}{{\text{m}}^3}$.

*f* is frequency. In this paper, microwave irradiation of $f=2.45{\text{GHz}}$ was used.

$\lambda$ is the wavelength, its unit is${\text{m}}$。.

${\varepsilon ^{\prime\prime}_{eff}}$ is affected by temperature and frequency.

${E_{rms}}$ is a function with temperature, frequency, and spatial coordinates, and strictly speaking, are all the functions with temperature^21,22^, so the temperature rise rate at this stage is a transcendental equation. Although it is difficult to give a quantitative analytical solution and generally needs to use the finite element method for numerical simulation to obtain the approximate solution, a basic qualitative analysis can be carried out. Based on this qualitative analysis and combined with the test results, the stage division of the physical process of microwave deicing on concrete pavement can be obtained.

The first stage is before the emergence of liquid water. The concrete absorbs the microwave and transfers heat to the ice. At the beginning of heating, $\lambda \ominus c\ominus \rho$、${\varepsilon ^{\prime\prime}_{eff}}$ and ${E_{rms}}$ are almost unchanged. ${\nabla ^2}{T_s}$gradually increases from 0; that is, the heat dissipation rate is accelerated, and the heat generation rate is almost unchanged. Therefore, the temperature rise rate decreases a little until a large amount of liquid water is generated after 0 ℃.

The second stage is from the formation of the water layer to the time when the water layer is basically completely formed, that is, from the time when the temperature is 0 ℃ until the temperature rise rate reaches the maximum value. The water layer gradually replaces the concrete as the wave-absorbing heat source. At this stage, the heat generation rate increases rapidly because, according to the formula of temperature rise rate of medium absorbing heat, the $fE_{{rms}}^{2}/c\rho$ of water is basically the same as that of concrete but ${\varepsilon ^{\prime\prime}_{eff}}$ is much higher than that of concrete (about 30 times that of concrete). Therefore, under the same conditions, the temperature rise rate of water is much higher than that of concrete; that is, with the increase of liquid water after 0 ℃, the heat dissipation rate is basically unchanged, and the heat generation rate increases sharply. As a result, the temperature rise rate of concrete surface increases.

The third stage is from the basic formation of the water layer to the ice breaking. The water layer absorbs microwave heating and transfers heat to concrete and ice. At this stage, the heat production rate is almost unchanged. That is because the water layer is basically formed, the thickness of water increases, and the area almost does not increase. According to the heat production rate formula, the heat production rate of water is almost unchanged. On the other hand, the heat dissipation rate of this stage increases gradually, mainly because the thickness of the ice layer decreases gradually, resulting in the heat conduction rate of water to the ice layer increasing gradually. Before the ice layer breaks, ${\text{d}}T/{\text{d}}z$ tends to infinity, and ${\nabla ^2}{T_s}$ tends to infinity, too. Then, the heat dissipation rate also tends to be infinity. Therefore, in general, the temperature rise rate decreases.

The fourth stage is after the ice breaking. The water layer directly conducts thermal convection with the air, and the heat dissipation rate is very slow. Therefore, the temperature rise rate after ice breaking increases. With the expansion of the ice-breaking area, the heat dissipation rate will approach the infinity value that appeared in the third stage, and the temperature rise rate will decrease and cycle, resulting in one step after another in the temperature rise curve at this stage. The temperature rise rate will not be stable until the ice-breaking area is stable and the ice layer is completely melted, and then, the curve will be similar to the state without the ice layer.

### Five evaluation indexes of pavement deicing technology

Based on experimental research and theoretical analysis, and based on the four-stage division theory of microwave deicing, which is the physical process of pavement concrete, five indexes can be used to evaluate the effect of pavement microwave deicing.

(1) ${\tau _0}$: The time when the temperature of the concrete surface reaches 0 ℃. The shorter, the better. The shorter the time spent to reach 0 ℃, the higher the temperature rise rate, and also, the shorter the deicing time will be. In 2008, Ding W et al.^23^ put forward the time when the ice pavement interface temperature reached 0 ℃ as the evaluation index. Through testing and simulation, they considered that the best time for microwave heating should be the time during the ice pavement interface temperature reached 0 ℃ and the melting of ice.

(2) ${\tau _1}$: The time when the water layer is fully formed. The shorter, the better. Since there is a process of “occurrence of water layer - complete formation of water layer” in the separation of the ice layer, the best time for deicing should be during this process. ${\tau _0}$, when the concrete surface reaches 0 ℃ is only the time when the water layer appears, and the ice layer begins to separate. At this time, the binding force between the ice layer and the concrete surface is still very large. However, as to ${\tau _1}$, as this time, the water layer is completely formed, and the ice layer is completely separated from the pavement, and the deicing effect would be much better.

(3) $\Delta \tau$: The time spent from the complete formation of water layer to ice breaking. The longer, the better. The occurrence of ice breaking indicates that a large amount of liquid water has been produced in the ice layer, and the water layer is in direct contact with the air, which is prone to secondary icing. The deicing time must be before the occurrence of ice breaking. Therefore, the longer it is, the slower the ice layer melts, the longer the mobility time left for the staff, the smaller the probability of secondary icing, and the better the deicing effect.

(4) ${S_0}$: The deicing area, which is obviously the larger the better.

(5) ${S_1}$: The ice-breaking area, which should be as small as possible.

### Effect of carbon fiber on deicing efficiency

According to the above five indexes, the deicing efficiency of four groups with different specimens (PC、0.1CFC、0.3CFC、0.6CFC) involved in this paper is counted in Table 1.

**Table 1:** Comparison of microwave deicing efficiency of concrete specimens with different carbon fiber lengths.

Unit	${\tau _0}$	${\tau _1}$	$\Delta \tau$	${S_1}$	${S_0}$	$\Delta T$
	s	s	s	cm^2^	cm^2^	℃
PC	60	116	none	none	125.59	16.8
0.1CFC	none	none	none	none	22.10	9.7
0.3CFC	52	112	8	large	149.12	24.2
0.6CFC	38	51	42	small	163.73	26.2

**Fig. 13:**
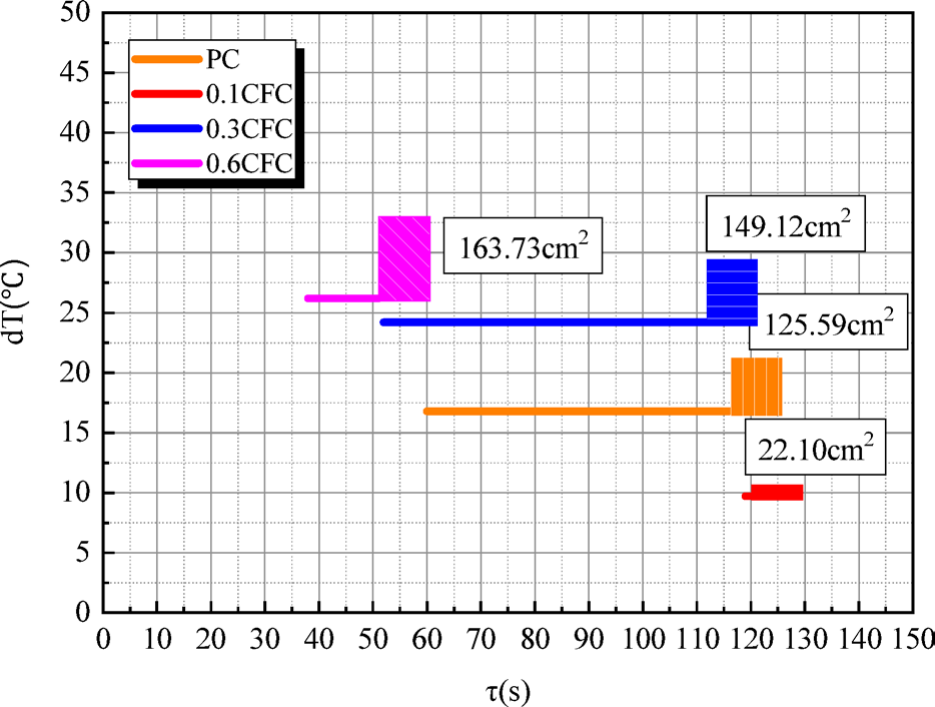
Line segment histogram of microwave deicing efficiency comparison.

For a more intuitive comparison, the table is represented by a “line segment histogram” (Fig. 13). In the figure, the horizontal axis of the coordinate represents the heating time, and the vertical axis represents the temperature rise amplitude of the central point T0 of the test piece after heating for 120s. The four line segments represent four groups of test pieces with different carbon fiber lengths. The starting point of the line segment is the time when the temperature at the central point T0 of the test piece surface reaches 0 ℃ (i.e., the water layer begins to appear), and the ending point is the time when the temperature rise rate begins to decrease (i.e., the water layer is completely formed), After the end point, the final deicing area is represented by the height of the histogram.

In general, 0.6CFC has high temperature rise range$\Delta T$, fast temperature rise rate, large deicing area ${S_0}$ and small ice breaking area ${S_1}$. Its deicing speed is 2.0 times that of PC, and the deicing area is 1.2 times that of PC. Compared with other specimens, 0.6CFC has the best deicing effect.

## Conclusions

(1) The physical process of microwave deicing of concrete pavement is a coupled process of multi-physical fields, and the equation for solving its temperature rise rate is a transcendental equation. The transcendence of microwave deicing is divided into four stages according to the characteristics of ice melting. The temperature rise rate of each stage can be qualitatively analyzed by the relative relationship between heat production and heat dissipation. This is of great significance in advancing the application of microwave deicing.

(2) Based on the comprehensive analysis of the experimental results, the deicing efficiency index system for evaluating the microwave deicing of concrete pavement in the laboratory or in the field is given. This helps to realize the quantitative evaluation and efficiency improvement of concrete microwave deicing.

(3) Adding carbon fibers to concrete can improve the microwave absorption efficiency of pavement concrete and thus improve the microwave deicing efficiency. Different lengths of carbon fiber reinforcement have different effects. Compared with PC, the deicing speed of 0.6 cm carbon fiber-modified concrete can be increased by 2.0 times, and the deicing area can be increased by 1.2 times.

(4) In the next step, we will conduct experimental studies based on pavement concrete engineering cases and discuss the energy consumption and cost-effectiveness of microwave deicing compared to conventional deicing methods, in order to enhance the practical application value of the research results.

## Data Availability

The data supporting this research result can be obtained from the Defense Engineering Laboratory of the Air Force University, but restrictions apply to the availability of these data, which were used under licence for the current study and so are not publicly available. The data are, however, available from the authors upon reasonable request and with the permission of Defense Engineering Laboratory of the Air Force University. The data availability statement specifies the point of contact as either the corresponding author or one of the co-authors.
